# Explainable machine learning for diffraction patterns

**DOI:** 10.1107/S1600576723007446

**Published:** 2023-09-20

**Authors:** Shah Nawaz, Vahid Rahmani, David Pennicard, Shabarish Pala Ramakantha Setty, Barbara Klaudel, Heinz Graafsma

**Affiliations:** a Deutsches Elektronen-Synchrotron DESY, Notkestraße 85, 22607 Hamburg, Germany; b Gdańsk University of Technology, Gdańsk, Poland; c Mid-Sweden University, Sundsvall, Sweden; SLAC National Accelerator Laboratory, Menlo Park, USA

**Keywords:** explainable machine learning, gradient-weighted class activation mapping, Grad-CAM, visualization of representations

## Abstract

Serial crystallography experiments at X-ray free-electron laser facilities produce massive amounts of data but only a fraction of these data are useful for downstream analysis. Thus, it is essential to differentiate between acceptable and unacceptable data, generally known as ‘hit’ and ‘miss’, respectively. Image classification methods from artificial intelligence, or more specifically convolutional neural networks (CNNs), classify the data into hit and miss categories in order to achieve data reduction. The quantitative performance established in previous work indicates that CNNs successfully classify serial crystallography data into desired categories [Ke, Brewster, Yu, Ushizima, Yang & Sauter (2018). *J. Synchrotron Rad.* **25**, 655–670], but no qualitative evidence on the internal workings of these networks has been provided. For example, there are no visualization methods that highlight the features contributing to a specific prediction while classifying data in serial crystallography experiments. Therefore, existing deep learning methods, including CNNs classifying serial crystallography data, are like a ‘black box’. To this end, presented here is a qualitative study to unpack the internal workings of CNNs with the aim of visualizing information in the fundamental blocks of a standard network with serial crystallography data. The region(s) or part(s) of an image that mostly contribute to a hit or miss prediction are visualized.

## Abstract 2

This article describes a qualitative study to unpack the internal workings of convolutional neural networks with the aim of visualizing information in the fundamental blocks of a standard network with serial crystallography data. Moreover, the study highlights region(s) or part(s) of an image that mostly contribute to a hit or miss prediction.

## 1. Introduction

Serial femtosecond crystallography (SFX) has become popular in determining biological structures from crystal diffraction patterns using X-ray free-electron laser (XFEL) sources (Chapman *et al.*, 2011 ▸; Wiedorn *et al.*, 2018 ▸). Using X-ray pulses, the experiments can produce strong patterns from weakly diffracting crystals at room temperature. However, these pulses also destroy the crystals, so diffraction patterns need to be gathered from many crystals. This results in large quantities of data. For example, the Coherent X-ray Imaging instrument at the Linac Coherent Light Source (LCLS, Menlo Park, California, USA) delivers full frames of data at up to 120 Hz, producing 43 000 samples per hour and data files of tens to hundreds of terabytes in size (Barty *et al.*, 2014 ▸).

**Figure 1:**
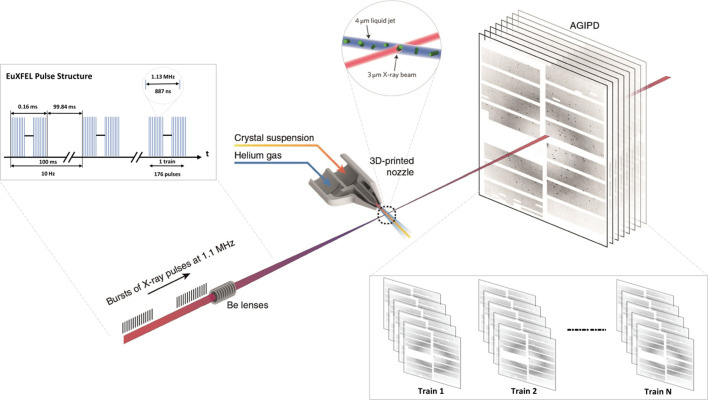
A typical SFX experiment at the European XFEL. The laser produces ten trains of X-ray pulses per second, with a pulse repetition rate within each train that can vary from 1.1 to 4.5 MHz. The diffraction from the protein sample is measured using an adaptive gain integrating pixel detector (AGIPD), which is capable of measuring up to 352 images from each bunch train at frame rates up to 4.5 MHz (Wiedorn *et al.*, 2018 ▸).

In spite of the large data volumes produced, only a small percentage of the data are useful for downstream analysis. Fig. 1 ▸ illustrates a typical experimental setup at the European X-ray Free-Electron Laser (European XFEL, Schenefeld, Germany). Protein crystals are fired through the path of the X-ray beam in a liquid jet, and only a small proportion of the X-ray pulses will actually hit a crystal. For example, an early SFX experiment at the European XFEL involving CTX-M-14 β-lactamase produced 3 215 616 images at an average rate of 300 images per second. Of these, only 14 445 images (0.4%) were observed to contain diffraction patterns from protein crystals as observed in the offline analysis (Wiedorn *et al.*, 2018 ▸). In this early experiment, both the accelerator and detector were operated below their maximum rates; at full speed, up to 3520 images per second can be taken. Furthermore, new free-electron laser facilities such as LCLS-II will handle experiments with continous repetition rates up to 1 MHz, resulting in even higher data volumes (Galayda, 2018 ▸).

**Figure 2:**
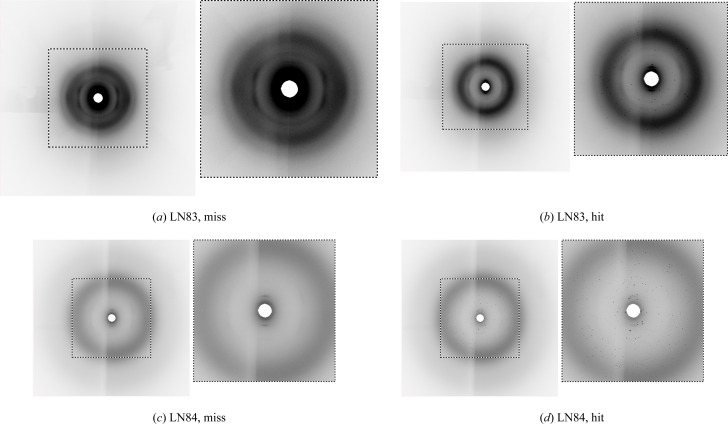
Representative diffraction patterns randomly selected from the Rayonix detector. We have cropped the central region of the diffraction patterns to enhance the visibility of the Bragg peaks in miss and hit categories. Ke *et al.* (2018 ▸) used human annotators to label data sets LN83 and LN84.

Considering these data challenges, sophisticated tools have been developed to process data and provide feedback during experiments (Barty *et al.*, 2014 ▸; White *et al.*, 2012 ▸; Daurer *et al.*, 2016 ▸; Winter *et al.*, 2018 ▸). In a typical SFX experiment, the detector may register Bragg peaks from crystal ‘hits’, otherwise missing in empty shots. Given the nature of SFX experiments, it is obvious that only samples with Bragg peaks are useful for downstream analysis (Wiedorn *et al.*, 2018 ▸). Fig. 2 ▸ shows ‘miss’ and ‘hit’ samples randomly selected from a Rayonix detector (Ke *et al.*, 2018 ▸).

Current methods utilize statistical peak finding to identify and discriminate between samples (Hadian-Jazi *et al.*, 2017 ▸, 2021 ▸). For example, the *Cheetah* software tool (Barty *et al.*, 2014 ▸) finds and counts the Bragg peaks in an image and keeps it if the counts exceed a certain threshold based on the Peakfinder8 algorithm. The threshold mechanism finds Bragg peaks with a size of more than *n*_min_ but fewer than *n*_max_ connected pixels with intensity values above a radially dependent threshold, which is calculated from the averaged background intensity. If the number of Bragg peaks with an adequately high signal-to-noise ratio surpasses a certain minimum number *n*_peaks_, the image is classified as a hit class. Finally, the reduced data are output in a facility-independent HDF5 format, enabling downstream analysis. For example, *CrystFEL* (White *et al.*, 2012 ▸) is employed to view, index, integrate, merge and evaluate diffraction data. Likewise, the *DIALS* software (Winter *et al.*, 2018 ▸) is extensively employed to detect Bragg peaks and index them. Other tools such as *OnDA* (Mariani *et al.*, 2016 ▸) and *Hummingbird* (Daurer *et al.*, 2016 ▸) provide real-time monitoring of data along with experimental conditions.

**Figure 3:**
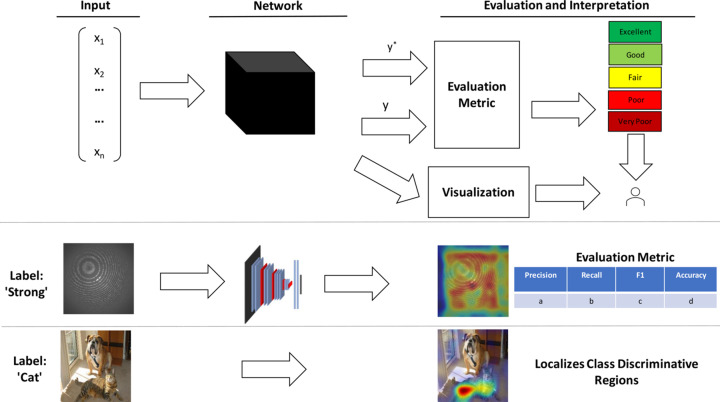
An overview of the proposed methodology to evaluate a deep neural model with interpretation and evaluation metrics. Visual interpretation provides useful information for experts to understand how a CNN classifies a sample into a certain class, while the evaluation metric helps to understand its quantitative performance. For illustration, the figure shows an example with a natural image, where discriminative regions in an image from the ‘cat’ class are highlighted.

Over the past decade, machine learning has produced unprecedented breakthroughs in various computer vision tasks (LeCun *et al.*, 2015 ▸). With these advancements, the crystallography community has also made use of machine learning for various applications (Sullivan *et al.*, 2019 ▸; Park *et al.*, 2017 ▸; Ryan *et al.*, 2018 ▸; Wang *et al.*, 2020 ▸). Specifically, the serial crystallography community has experimented with these methods to achieve data reduction (Becker & Streit, 2014 ▸; Ke *et al.*, 2018 ▸; Souza *et al.*, 2019 ▸; Rahmani *et al.*, 2023 ▸; Chen *et al.*, 2021 ▸). Machine learning, or more specifically deep learning methods including convolutional neural networks (CNNs), encode experimental data to classify it into hit or miss categories. While these networks can achieve superior performance on image classification tasks, their lack of decomposability into individually intuitive blocks makes them hard to understand or interpret (Lipton, 2018 ▸). Previous work with CNNs for serial crystallography has inherited these limitations (Ke *et al.*, 2018 ▸). For example, there is no mechanism to explain how CNNs make decisions while classifying samples into hit or miss categories. Generally, input data in a CNN pass through several layers of multiplication with learned weights and through nonlinear transformations in order that a prediction can be made. Therefore, a prediction may involve millions of mathematical operations depending on the network configuration. This process makes it challenging for humans to understand the exact mapping from input to prediction – it is a ‘black box’ (Fig. 3 ▸). As a result, when such networks fail, they often break down spectacularly without providing meaningful error explanations (Lipton, 2018 ▸). Therefore, these networks should provide visualizations on their predictions along with standard evaluation metrics.

To this end, the computer vision community has developed methods to explain model predictions visually (Mahendran & Vedaldi, 2015 ▸; Selvaraju *et al.*, 2017 ▸; Simonyan *et al.*, 2014 ▸). Similarly, the crystallography community also uses these visualization methods, such as class activation maps, to highlight areas with Bragg peaks (Chen *et al.*, 2021 ▸). Continuing this process, we present here a comprehensive study exploiting visualization methods from computer vision to highlight data features (attributes) that contribute to the CNN prediction or decision in classifying serial crystallography data into hit or miss categories, using both synthetic and real experimental data. In addition, we use computer vision methods to understand what information different layers of a CNN extract from the image. Our qualitative examples reveal that a CNN focuses on discriminative regions of an image while classifying data into hit or miss categories. In other words, it activates different parts for images taken from miss or hit classes.

## 2. Method

Our goal is to understand, with visualization, how CNNs classify serial crystallography data into hit and miss categories. Fig. 3 ▸ shows our proposed methodology employing both qualitative and quantitative components. In this work, we use two computer vision methods to visualize the function of CNN representations and CNN predictions. Generally, visualization methods are applied to supervised neural networks. Therefore, we selected the standard image classification networks named AlexNet (Krizhevsky *et al.*, 2012 ▸) and Residual Network (ResNet) (He *et al.*, 2016 ▸) to provide insights into internal workings. In this section, we provide details of various components of our proposed methodology, including data, supervised neural network details and visualization methods, along with implementation details.

### 2.1. Data sets

Previous work involving machine learning has used both synthetic and experimental data sets (Ke *et al.*, 2018 ▸; Souza *et al.*, 2019 ▸; Rahmani *et al.*, 2023 ▸). Thus, we selected data sets to visualize CNN representations along with the parts responsible for a certain prediction. The DiffraNet data set is composed of synthetic samples generated using the *nanoBragg* simulator (Souza *et al.*, 2019 ▸; https://bl831.als.lbl.gov/~jamesh/nanoBragg/). The simulator produces different images by taking a single-crystal structure and varying the X-ray beam intensity, simulating imperfections in the crystal by breaking it up into smaller crystals, and also by varying parameters like the sources of background noise and the orientation of the crystal. DiffraNet consists of 25 000 samples with an image size of 512 × 512 divided into five classes: Blank, No crystal, Weak, Good and Strong. The Blank class denotes images with no X-rays and only detector noise, while in the No crystal class there is scattering from amorphous material but no protein crystal. Weak, Good, and Strong represent images with a crystal in the beam with increasingly higher intensity.

We also used real experimental data sets (LN83 and LN84) collected on the Macromolecular Femtosecond Crystallography (Boutet *et al.*, 2016 ▸) instrument of the LCLS (White *et al.*, 2015 ▸) with conveyor-belt delivery of crystal specimens (Fuller *et al.*, 2017 ▸). Previous work (Ke *et al.*, 2018 ▸) unpacked the first 2000 images from the native LCLS data format for further study.

Table 1 ▸ shows the experimental settings for these two data sets. We have used the same images and labels as in the experiments (Ke *et al.*, 2018 ▸). We note that the patterns labelled as hits by the human annotator are spot-finder hits having visible Bragg peaks. The indexable patterns would be only a subset of these patterns. Ke *et al.* (2018 ▸) curated these data sets to find a rapid screen for Bragg peaks so that non-hits could be vetoed before they ever hit flash memory, for example by implementing the CNN on a field-programmable gate array (FPGA) or a graphical processing unit (GPU). To this end, we fed LN83 and LN84 into the *DIALS* software tool to find out if the labelled hit patterns are indexable. We observed that 90% of the labelled hit patterns from LN83 and LN84 are indexable.

### 2.2. Convolutional neural networks

In recent years, deep neural models have made remarkable improvements in state-of-the-art image, video, speech and text processing tasks (LeCun *et al.*, 2015 ▸; Saeed *et al.*, 2022 ▸; Nagrani *et al.*, 2017 ▸; Mikolov *et al.*, 2013 ▸). One of the fundamental tools leading to these remarkable results is a network named the convolutional neural network (CNN). Typically, it is composed of several building layers to transform one volume of activations to another through a differentiable function. We provide a brief overview of three layers in a typical CNN, *i.e.* the convolution layer, the pooling layer and fully connected layers.

A *convolutional layer* extracts features from the input image or previous layer using the mathematical operation of convolution between the input and a filter of a particular size. The operation is performed with a sliding window over the input image and computes the dot product between the filters and the parts of the input with respect to the size of the filter, producing feature maps. These feature maps provide information on where features such as edges, corners *etc.* occur in the image and how well they correspond to the filter. The weights of the filters can be trained using gradient-descent-based algorithms such as stochastic gradient descent. The linear operation of convolution is then followed by applying a nonlinear activation function to each element of the feature map; this makes it possible for the network to learn nonlinear features of the input.

A convolutional layer is often followed by a *pooling layer* which shrinks the size of the convolved feature map to reduce the computational costs. In other words, the pooling operation keeps the detected features in a smaller representation by discarding less significant data at the cost of spatial resolution. The pooling task independently operates on each feature map. The most common methods are max and average pooling, where the former finds the highest value within a window region and discards the remaining values, while the latter finds the mean of the values within the region.

A series of convolutional and pooling layers extract increasingly high-level features of the image. These are followed by *fully connected layers* of neurons which perform a classification task. The final fully connected layer has one output node for each class.

**Figure 4:**
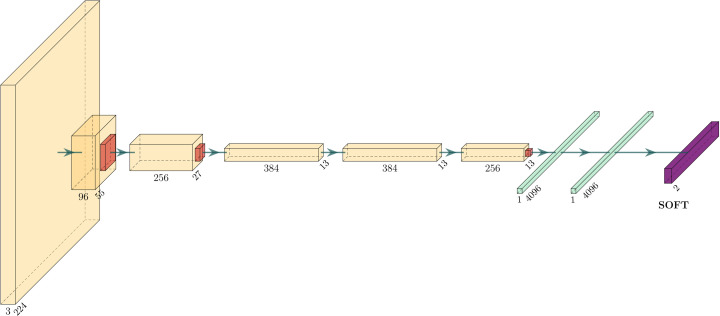
The AlexNet architecture. It contains eight layers; the first five being convolutional layers and the last three fully connected layers. The final fully connected layer has the same number of outputs as the number of classes in the data set.

In this work, we used a standard network named AlexNet (Krizhevsky *et al.*, 2012 ▸). Although it is one of the earlier neural networks, it has all the necessary fundamental components which are required for the two visualization methods. For example, it has both convolutional and fully connected layers. The network has eight layers, which is fewer than many more recent architectures, but it is still considered a deep neural network. The first five layers are convolutional layers, some of them followed by max-pooling layers, and the last three are fully connected layers, as shown in Fig. 4 ▸. Moreover, it uses the rectified linear unit (ReLU) activation function which is computationally cheap and widely used in CNNs.

We also employed a more recent and popular network known as ResNet (He *et al.*, 2016 ▸). The network introduces residual blocks to alleviate the vanishing gradient problem (generally, the performance of CNNs can be improved by stacking more layers, but it has been observed that after some depth the performance deteriorates) (Bengio *et al.*, 1994 ▸; Glorot & Bengio, 2010 ▸). The residual blocks consist of a skip connection which hops some layers in between. These skip connections allow the CNN to learn the identity functions, which ensures that the later layer will perform at least as well as the initial layer. Typically, ResNets can have variable sizes, depending on how big each of the layers are; we trained ResNet-101 in our experiments.

### 2.3. Visualization of representations

**Figure 5:**
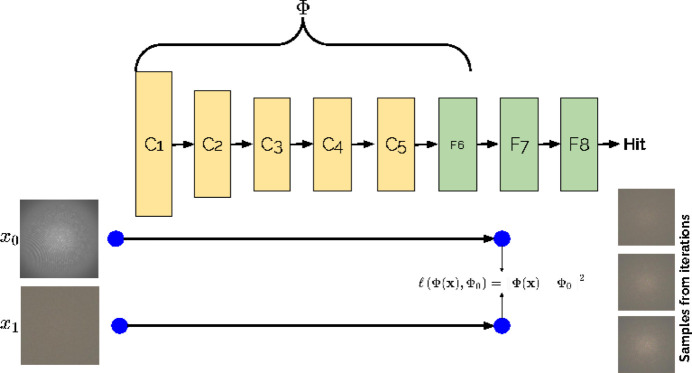
An overview of the process of visualizing CNN representations, prepared using the method of Mahendran & Vedaldi (2015 ▸). The method starts with random noise and iteratively reconstructs an equivalent image, demonstrating the CNN representations.

Image representations are a crucial component of almost any image-understanding system. They provide information to understand what is encoded by a CNN layer. This is done by taking the output of a CNN layer (referred to as representations) and attempting to reconstruct the original input image from it. Later CNN layers contain increasingly high-level information, so we do not expect an accurate or detailed reconstruction, but the reconstruction can indicate what features a layer retains. Fig. 5 ▸ shows an overview of the process to reconstruct the original image from the CNN representations. Mathematically, it is formulated as follows: 

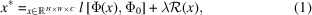

where Φ(*x*) refers to the representations obtained by passing some image *x* to the network, and Φ(*x*_0_) = Φ_0_ are the representations obtained from the original image. 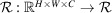 is a regularizer capturing an image prior, which is helpful for the reconstruction process by restricting the inversion to the subset of images. Intuitively, it produces an image x* that is similar to *x*_0_ from the representation view point. The process ensures that the output is some kind of reasonable image, not just computational noise. The loss function *l* employed in this work is the Euclidean distance,

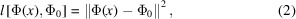

which minimizes the distance between the representations of the original image and reconstructed image. In addition, the regularizer improves the reconstruction process with the help of two image prior methods. The first prior used is called the α-norm, which is defined as

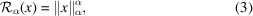

where *x* is the vectorized and mean-subtracted image. It favours images with a narrower spread of pixel values. The second prior used for a discrete image *x* is called the total variation, defined as

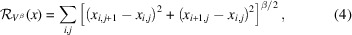

where β = 1. This favours images in which neighbouring pixels have similar values. Finally, the overall objective function is 

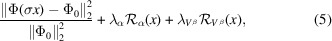

where the scaling σ is the average Euclidean norm of natural images in a training set and λ_α_ is the α-norm to encourage the reconstructed image σ*x* to be contained in a natural range.

As explained in Section 2.2, typically a CNN detects edges from pixels in the first layer(s), then uses those edges to detect shapes in the next layer(s), and then uses that result to infer complex shapes and objects in higher or later layers. Thus, the reconstructed image from the initial layers may look very similar to the original image (Mahendran & Vedaldi, 2015 ▸).

### 2.4. Visual explanations from predictions

Visual explanations or interpretations are employed by CNNs to highlight features (attributes) that mostly contribute to a specific prediction. For example, visual explanations can show the part(s) or region(s) of an image responsible for a ‘dog’ prediction with a model trained on dog and cat samples. Likewise, our goal is to visualize the part(s) of serial crystallography images responsible for hit or miss classifications. To this end, we use gradient-weighted class activation mapping (Grad-CAM) which requires a differentiable layer, generalizing it for a wide variety of CNN architectures (Selvaraju *et al.*, 2017 ▸).

Grad-CAM takes the feature map of the last convolutional layer and multiplies every channel by the gradient of the output class. A heat map is then generated to highlight the activated region(s) of the input image for a specific class. We use the following steps to create heat maps for visual explanations on a specific prediction:

(i) Compute the gradient of the score for a specific class *y* ^*c*^ (the raw output of the last convolutional layer before softmax, which is a mathematical function that converts a vector of *K* real numbers into a probability distribution of *K* possible outcomes) with respect to each of the feature map activations (*A* ^*k*^) of a convolutional layer.

(ii) Average-pool the gradients over the width and height dimensions to get the neuron importance weights ( 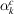 ). This gives us a measure of how strongly each feature map (*k*) contributes to an image being classified as a particular class (*c*),

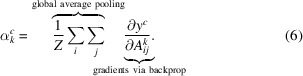




(iii) Calculate the heat map by finding the sum of all the feature maps, weighted by their importance, and follow it by a ReLU to obtain 

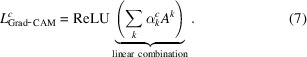

ReLU is applied to the linear combinations which have a positive influence on the specific class, suppressing negative pixels belonging to other classes.

(iv) Reshape and project the maps onto the original image.

(v) Finally, the merged original image and heat map highlight the discriminative region(s) contributing to the classification process.

### 2.5. Implementation details

We used a standard set of hyperparameters to train the AlexNet and ResNet-101 networks in all experiments. Specifically, the networks were trained on a GPU for 50 epochs using a batch size of 128 with an Adam optimizer (Kingma & Ba, 2014 ▸) having an exponentially decaying learning rate (initialized to 10^−5^). We followed the same train and test splits as used in previous work (Ke *et al.*, 2018 ▸; Souza *et al.*, 2019 ▸). We evaluated the performance of the networks with standard classification evaluation metrics, *i.e.* accuracy, precision and recall, defined as follows: 

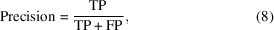




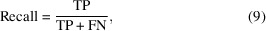




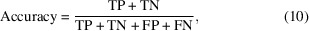

where TP is true positive, TN is true negative, FP is false positive and FN is false negative.

## 3. Experiments

In this section, we provide an overview of the data sets and implementation details along with qualitative and quantitative results. In our experiments, we visualized the CNN representations along with the region(s) important for making a specific prediction. The aim of these experiments was to understand the internal working of a standard CNN (AlexNet and ResNet-101) while classifying serial crystallography data.

In the first experiment, we trained the standard CNNs AlexNet and ResNet-101 on the simulated DiffraNet data set (Table 2 ▸). Our implementation produced competitive quantative performance compared with DeepFreak (Souza *et al.*, 2019 ▸). We also used a confusion matrix to summarize the prediction results on AlexNet (Table 3 ▸). We observed that misclassification often occurs between Weak and Good and Good and Strong categories. These results indicate that neighbouring classes are similar. Our implementation achieves perfect quantitative performance on Blank and No crystal categories. For the purposes of data reduction, which is the main aim, we do not care about Weak versus Good versus Strong, as long as we get hit versus non-hit correct. Thus, we can discard patterns from the Blank and No crystal categories, achieving meaningful data reduction. The quantitative performance (accuracy) indicates that the model is successfully classifying the synthetic images. Similarly, we performed the classification experiments with experimental data consisting of two diverse data sets denoted LN83 and LN84 (Table 4 ▸). Our implementation produced slightly lower accuracy than the previous work (Ke *et al.*, 2018 ▸).

Table 5 ▸ provides a detailed comparison between deep learning (Ke *et al.*, 2018 ▸) and automatic spot-finding methods (*DIALS* spot finding; Winter *et al.*, 2018 ▸), and Table 6 ▸ lists the *DIALS* parameters used to identify peaks or spots in the pattern. The traditional spot-finding method reliably discards miss patterns, but it does also throw away some hit patterns which are useful for downstream tasks such as indexing. On the other hand, deep learning methods reliably differentiate between hit and miss classes.

Evaluation numbers from previous work do not provide insight on how a CNN classifies images from serial crystallography. Thus, we provide qualitative insights with the following two experiments:

(i) Analyse CNN representations of various layers with the serial crystallography data. In other words, our goal is to analyse the information contained in a convolutional layer for distinct classes, for example Strong and Blank.

(ii) Visualize the input images while classifying the serial crystallography data into hit or miss categories. In other words, our goal is to analyse which regions are activated for distinct classes in serial crystallography data.

**Figure 6:**
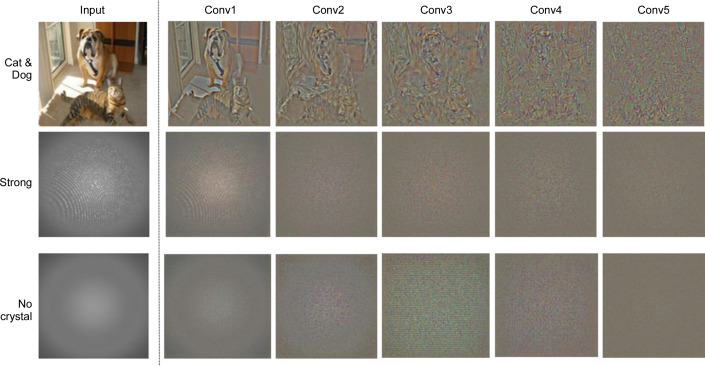
Three examples of CNN representations at the first five convolutional layers of AlexNet. The network detects edges from pixels in the first layer, then uses those edges to detect shapes in the next layer, and then uses that result to infer complex shapes and objects in later layers. Thus, despite growing fuzziness, convolutional layers continue to maintain photographically accurate representations of input images. (Best viewed in colour and enlarged.)

**Figure 7:**
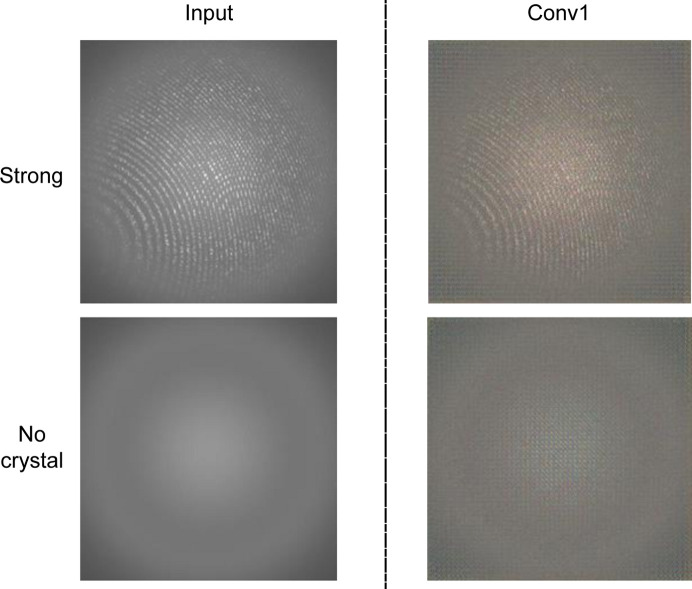
CNN representations of the first convolutional layers of AlexNet with two distinct classes (Strong and No crystal). The visual representations indicate that the network extracts uniquely different representations for the two classes.

We extracted representations from each layer (Conv1, Conv2, Conv3, Conv4, Conv5) of AlexNet. These representations were then used to visualize the information for serial crystallography data. For example, Fig. 6 ▸ shows a representation reconstructed from distinct classes (Strong and No crystal). We have included a natural image example in order to understand the qualitative visualizations. The first few layers are significantly similar to the input images because a CNN (AlexNet) detects edges from pixels in the first layer (Conv1) (Mahendran & Vedaldi, 2015 ▸). Thus, the first couple of layers maintain a faithful copy of the input image (Fig. 7 ▸).

We observe that all convolutional layers maintain a photographically faithful representation of the input image, although with increasing fuzziness. Likewise, the natural image also maintains similar representations (Mahendran & Vedaldi, 2015 ▸). The reconstructed representations from the Strong and No crystal images are dissimilar, indicating that the model extracts unique representations for each of them. We can conclude that our trained network learns different representations for each class.

**Figure 8:**
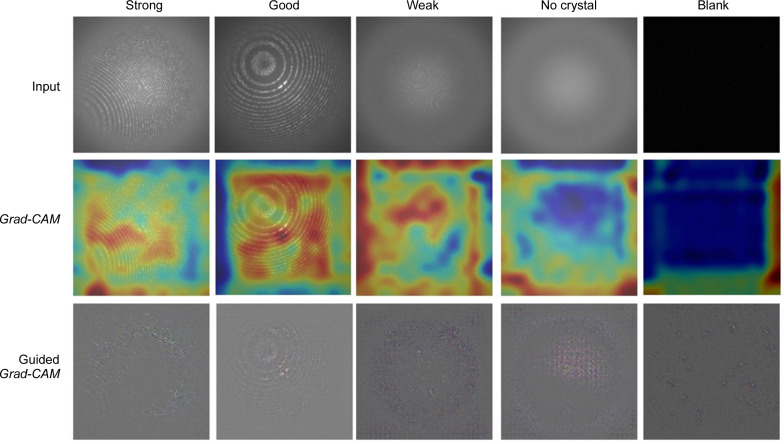
Grad-CAM visualization of five classes of DiffraNet, highlighting contributing features. Guided Grad-CAM images are included to highlight fine-grained details. (Best viewed in colour and enlarged.)

**Figure 9:**
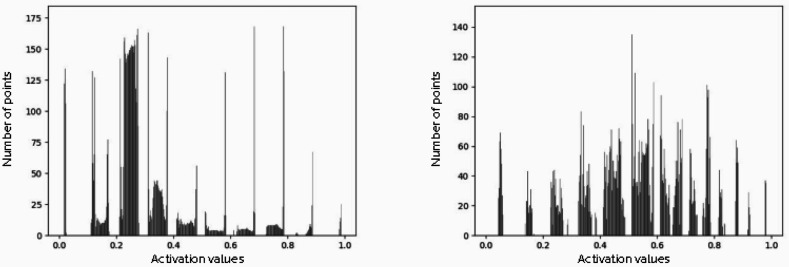
Histograms of the activation values. These values extracted randomly selected samples representing (left) Blank and (right) Strong classes in the DiffraNet data set.

**Figure 10:**
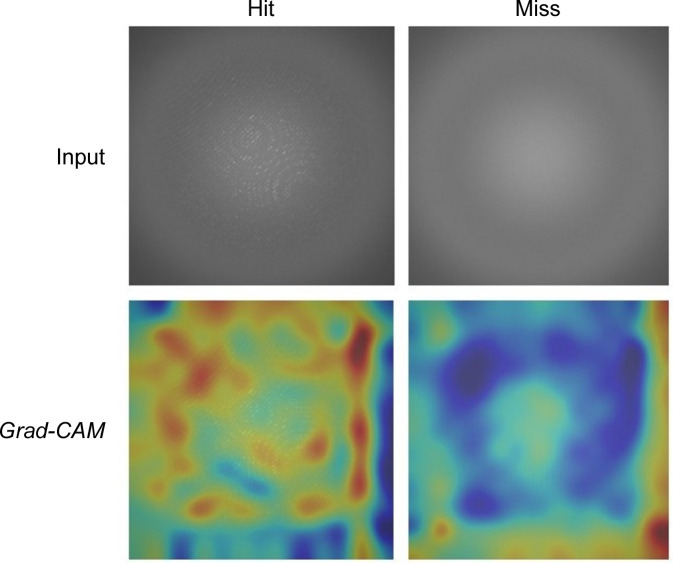
Grad-CAM and guided Grad-CAM visualizations from AlexNet, highlighting contributing features. (Best viewed in colour and enlarged.)

In the second set of experiments, we visualized the input images of different classes to analyse the regions or parts that contribute to a hit or miss prediction. Fig. 8 ▸ utilizes heat maps to focus on the regions that are activated while making a prediction with AlexNet. Heat maps generated with Grad-CAM show that the network localizes distinct regions while classifying the input images into Strong, Good, Weak, No crystal and Blank categories. We have added a guided Grad-CAM visualization to provide fine-grained details like pixel-space gradient visualization. We observe that the activations or focused areas are increased from Blank to Strong classes, indicating the presence of Bragg peaks (see Fig. 9 ▸). In another experiment, we merged Blank and No crystal classes into miss, and Weak, Good and Strong classes into hit, to train AlexNet with the aim of visualizing discriminative regions (Fig. 10 ▸). These visualizations show that the network focuses on distinct regions encompassing Bragg peaks while making a prediction.

**Figure 11:**
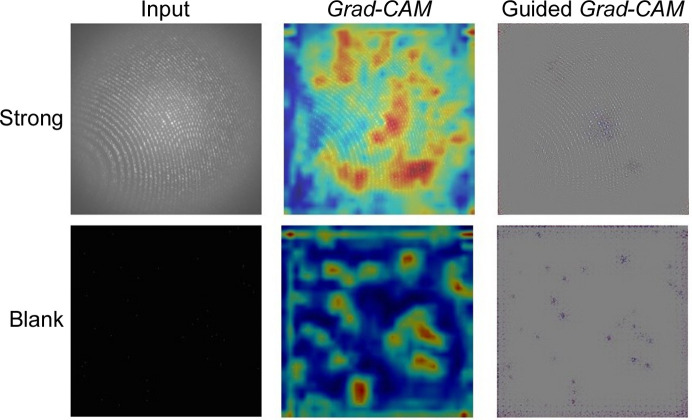
Grad-CAM and guided Grad-CAM visualizations from ResNet-101, highlighting contributing features. (Best viewed in colour and enlarged.)

**Figure 12:**
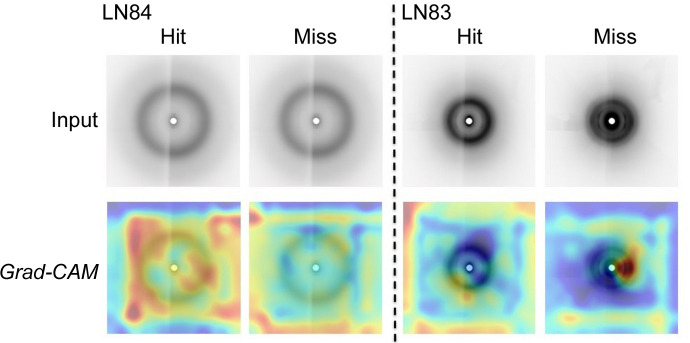
Grad-CAM and guided Grad-CAM visualizations with experimental data sets LN83 and LN84, highlighting contributing features. (Best viewed in colour and enlarged.)

We trained ResNet-101 with the aim of visualizing regions of different classes across other networks. Fig. 11 ▸ shows the heat map of activation values extracted from layer 4, a common practice to visualize regions with Grad-CAM and guided Grad-CAM. We observe that ResNet-101 activates regions encompassing Bragg peaks while making a prediction, similar to AlexNet, although the activations vary slightly for the two networks. However, these are generally increased from miss to hit classes, indicating the presence of peaks. Finally, we visualized discriminative regions for the experimental data sets LN83 and LN84. We selected random samples from the hit and miss categories (Fig. 12 ▸). We observe that the hit image contains higher activations than a miss for experimental data sets LN83 and LN84.

Our qualitative results indicate that the network encodes both the Bragg peaks and background of the input samples. Interestingly, a CNN focuses on regions of the image where Bragg peaks are present, if there are any. DiffraNet contains synthetic data with a high number of Bragg peaks which made the regions more prominent in our visualization. However, experimental data such as LN83 and LN84 contain fewer visible Bragg peaks, resulting in fewer activated regions. Our results indicate that the number of visible Bragg peaks affects the performance of the network along with the visualization (Table 4 ▸ and Fig. 12 ▸). Interestingly, Ke *et al.* (2018 ▸) used threshold values of 14 and 29 for LN83 and LN8, respectively, to classify the data as hit or miss. We observe that the hit and miss data are similar in nature when the number of Bragg peaks is smaller, which in turn affects the performance of the network.

## 4. Conclusion

In recent years, massive amounts of experimental data have been produced in serial femtosecond crystallography at X-ray free-electron laser facilities. Although these data sets are large, only a fraction of the data are useful for later analysis. There has thus been interest in using convolutional neural networks to process serial crystallography data. CNNs successfully categorize these data into the desired categories (hit and miss), but previous work has not explained how these networks achieve such results, making them a ‘black box’. We have presented here a qualitative and quantitative study to visualize the representations and discriminative regions significant to classifying serial crystallography data. Our study reveals that our trained networks encode both Bragg peaks and background to classify these test image sets into hit or miss categories.

## Figures and Tables

**Table 1:** Experimental data

LCLS data set (proposal, run)	Incident energy (eV)	Protein	Space group, unit cell (Å)	Instrument	Sample delivery	Detector
LN84, 95	9516	Photosystem II	*P*2_1_2_1_2_1_, *a* = 118, *b* = 223, *c* = 311	MFX	Conveyor belt	Rayonix
LN83, 18	9498	Hydrogenase	*P*2_1_2_1_2_1_, *a* = 73, *b* = 96, *c* = 119	MFX	Conveyor belt	Rayonix

**Table 2:** Classification results with AlexNet and ResNet-101 on DiffraNet data sets GLCM is the grey-level co-occurrence matrix, ORB is the oriented FAST and rotated BRIEF feature extractor, and MLP is a multilayer perceptron. The best result is shown in bold.

Method	Accuracy
DeepFreak (GLCM+random forest) (Souza *et al.*, 2019 ▸)	98.4
DeepFreak (GLCM+support vector machine) (Souza *et al.*, 2019 ▸)	97.6
ORB+MLP (Rahmani *et al.*, 2023 ▸)	97.5
DeepFreak (Souza *et al.*, 2019 ▸)	**98.8**
AlexNet (our implementation)	98.1
ResNet-101 (our implementation)	98.3

**Table 3:** DiffraNet confusion matrix for the test set with AlexNet

		Blank	No crystal	Weak	Good	Strong	Recall (%)
True class	Blank	2069	0	0	0	0	100
	No crystal	2	3266	0	0	0	99.9
	Weak	3	24	3273	46	0	97.8
	Good	0	0	62	2341	41	95.8
	Strong	0	0	0	60	1412	95.9

Precision (%)		99.9	99.3	98.1	95.7	97.1	

**Table 4:** Classification results (accuracy) on real experimental data sets with AlexNet and ResNet-101 The best results are shown in bold.

	Data sets	
Method	LN83	LN84
Ke *et al.* (2018 ▸)	**96.0**	**90.0**
AlexNet (our implementation)	82.2	87.0
ResNet-101 (our implementation)	90.4	91.2

**Table 5:** Comparison between deep learning [AlexNet and ResNet-101 in the present work, and the results of Ke *et al.* (2018 ▸)] and an automatic spot-finding method (Winter *et al.*, 2018 ▸)

		AlexNet (present work)		ResNet-101 (present work)		Ke *et al.* (2018 ▸)		Spot finding	
Data set	Human expert	Hit or maybe	Miss	Hit or maybe	Miss	Hit or maybe	Miss	Hit or maybe	Miss
LN83	Hit or maybe	75.5	24.5	85.6	14.4	98.5	1.5	85.8	14.2
	Miss	15.2	84.8	6.5	93.5	3.1	96.9	0.1	99.9

LN84	Hit or maybe	95.9	4.1	93.0	3.0	98.5	1.5	69.9	30.1
	Miss	20.0	80.0	7.2	92.8	10.1	89.9	0.4	99.6

**Table 6:** Automatic spot-finding parameters (Ke *et al.*, 2018 ▸)

Data set	Gain (ADU photon^−1^)	Global threshold (ADU)	σ_Strong_	Minimum spot area (pixels)	Wall clock time (s), 16 processors
LN83	0.27	200	3	3	80.3
LN84	0.31	200	3	3	89.3
